# Correction: Independent regulation of mitochondrial DNA quantity and quality in *Caenorhabditis elegans* primordial germ cells

**DOI:** 10.7554/eLife.93941

**Published:** 2023-11-01

**Authors:** Aaron ZA Schwartz, Nikita Tsyba, Yusuff Abdu, Maulik R Patel, Jeremy Nance

**Keywords:** *C. elegans*

Schwartz AZA, Tsyba N, Abdu Y, Patel MR, Nance J. 2022. Independent regulation of mitochondrial DNA quantity and quality in *Caenorhabditis elegans* primordial germ cells. *eLife* **11**:e80396. doi: 10.7554/eLife.80396.

Published 6 October 2022

It has come to our attention that a few clerical errors were made in Excel during figure preparation that resulted in minor errors in the manuscript. Specifically, technical replicate data was unintentionally omitted from three calculations. None of the outcomes or conclusions of the manuscript are affected by these corrections. We sincerely apologize for the oversight.

Figure 1F: Technical replicate data points were excluded from calculating the mean of proportional PGC cell body mitochondria in 1.5-fold and 2-fold embryos. Originally reported: 0.1998+- .01 and 0.3754+-0.01. Corrected: 0.1898+-0.001 and 0.3801+-0.01 respectively, ultimately increasing the statistical significance of this comparison slightly: originally p<0.0006, now p<0.0001. The associated figure, legend, and source data file are corrected (bolding indicates change or addition).

Corrected Figure 1 figure legend: "... and the S.E.M as error bars. **P≤0.01, ***P≤0.001, ****P≤0.0001, unpaired two-tailed Student’s *t*-test..."

Original Figure 1 figure legend: "... and the S.E.M as error bars. **P≤0.01, ***P≤0.001, unpaired two-tailed Student’s *t*-test..."

Figure 1 - Figure supplement 2C: Technical replicate data points were excluded when calculating the means of whole embryo mtDNAs, which has been corrected. This change had no net effect on statistics or analysis, but had a minor effect on the reported values. This was corrected in the text, associated figure, and source data file.

Corrected text: "...the number of mtDNAs we detected in whole early embryos (**33,840±1784**)..."

Original text: "...the number of mtDNAs we detected in whole early embryos (**33,875±1819**)..."

Corrected text: "...*TFAM-GFP* knock-in strain contained significantly fewer mtDNAs (**8450±768**) than wild type..."

Original text: "...*TFAM-GFP* knock-in strain contained significantly fewer mtDNAs (**8630±662**) than wild type..."

In Figure 2F: Only two of three technical ddPCR replicates were used to calculate the means for *TFAM-GFP* PGC mtDNAs. This was corrected in the text, associated figure and source data file, resulting in a slight increase in statistical significance of this comparison: originally *P*<0.0025, corrected *P*<0.0005.

Corrected text: "...The *TFAM-GFP* embryonic PGCs contained **89±3** mtDNAs and *TFAM-GFP* L1 PGCs contained **54±2** mtDNAs..."

Original text: "...The *TFAM-GFP* embryonic PGCs contained **94±5** mtDNAs and *TFAM-GFP* L1 PGCs contained **56±2** mtDNAs..."

Corrected Figure 2 figure legend:"...and the SEM as error bars. n.s., not significant (*P*>0.05), *P≤0.05, ***P≤0.001, unpaired one-tailed..."

Original Figure 2 figure legend:"...and the SEM as error bars. n.s., not significant (*P*>0.05), *P≤0.05, **P≤0.01, ***P≤0.001, unpaired..."

Additionally, as a result of copying error, Figure 6 - source data 1 had duplicate data presented for *pdr-1;uaDf5* heteroplasmy resulting in inaccurate calculation of the mean value for 3/17/22, original, 64.0 (duplicated from 3/10/22), corrected 67.7; and an error in the reported mean for *pink-1* mtDNA proportion: original 0.7567+–0.1, corrected 0.752+–0.1. Both were only present in the source data file, and did not affect Figure 6. We have included a corrected source data file.

The corrected Figure 1 is shown here:

**Figure 1:**
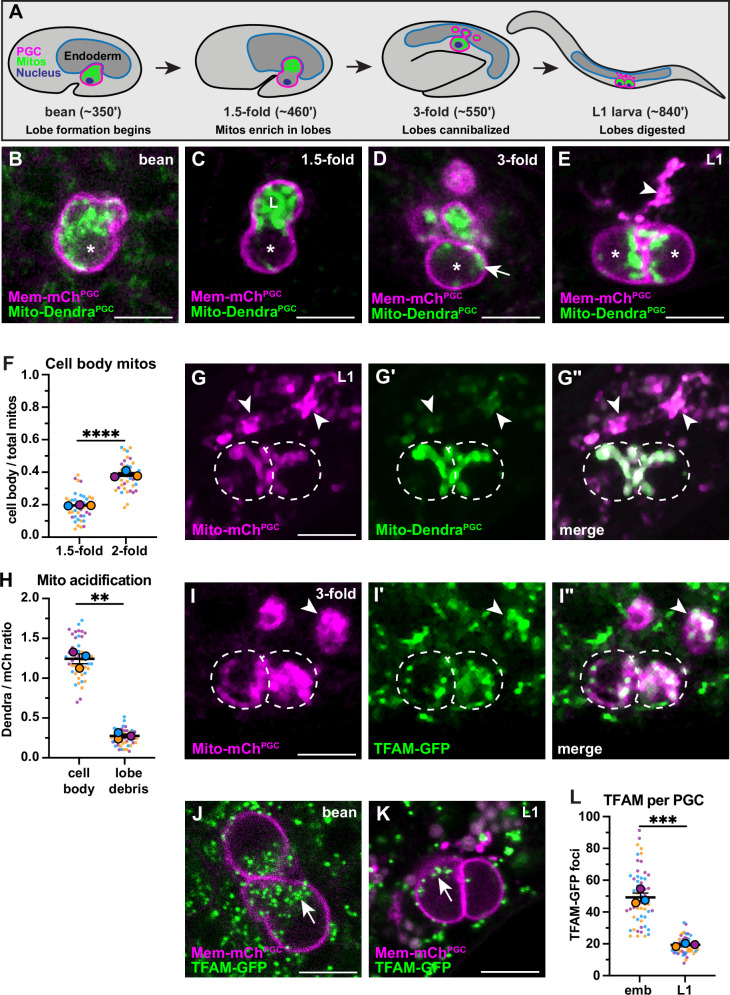


The originally published Figure 1 is shown for reference:

**Figure 2:**
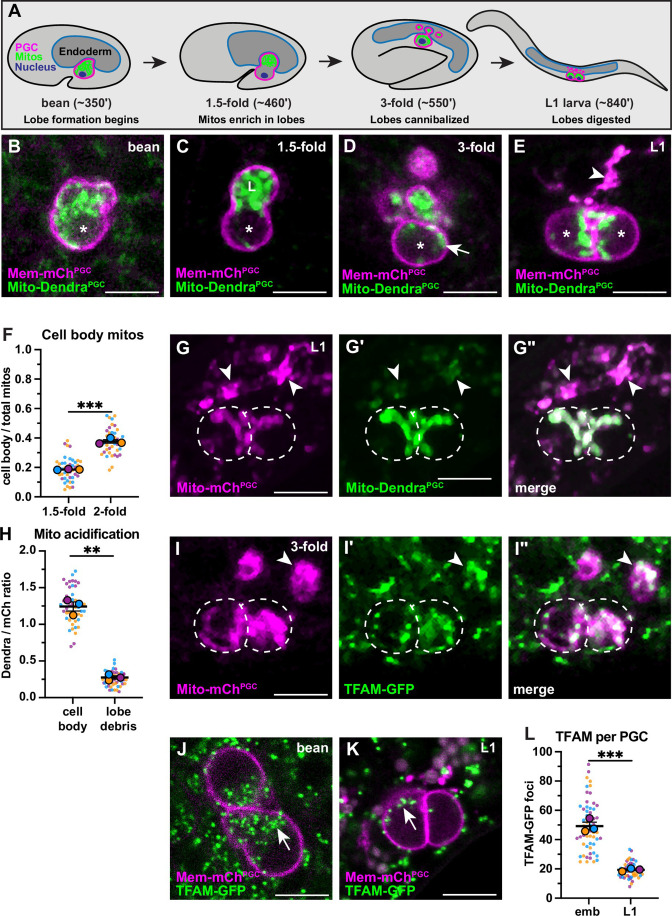


The corrected Figure 1- figure supplement 2 is shown here:

**Figure 3:**
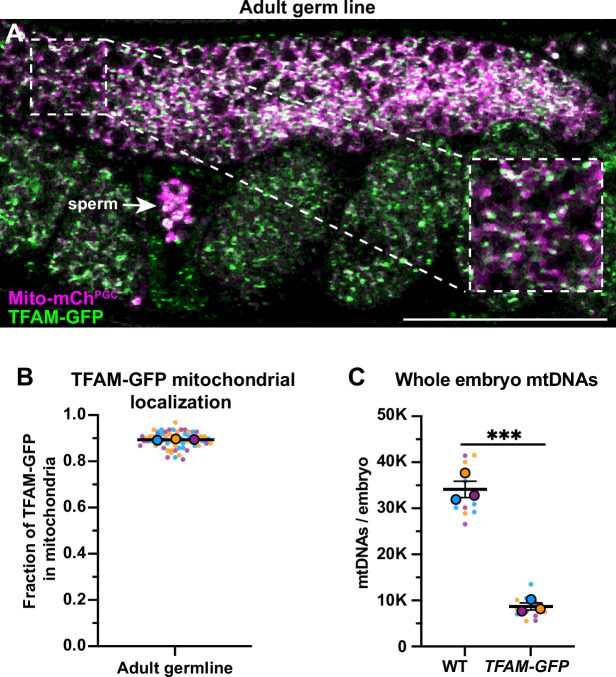


The originally published Figure 1- figure supplement 2 is shown for reference:

**Figure 4:**
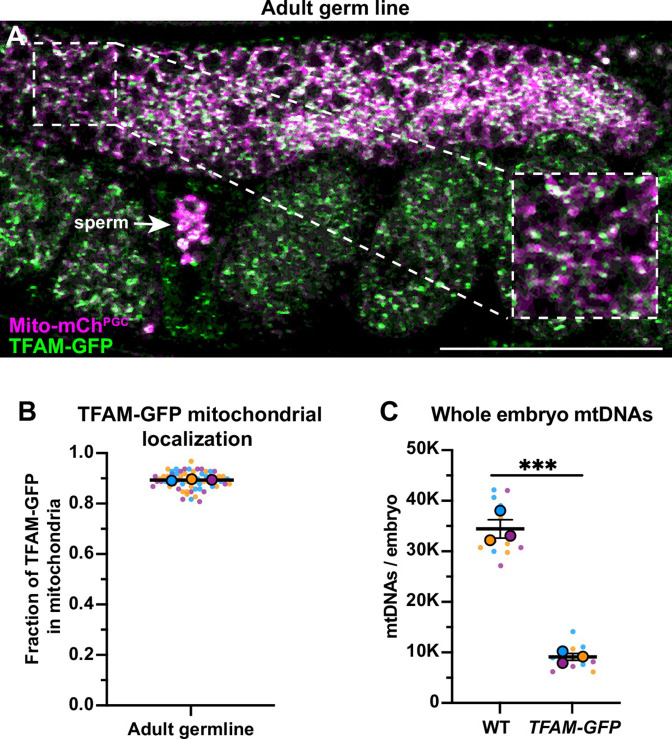


The corrected Figure 2 is shown here:

**Figure 5:**
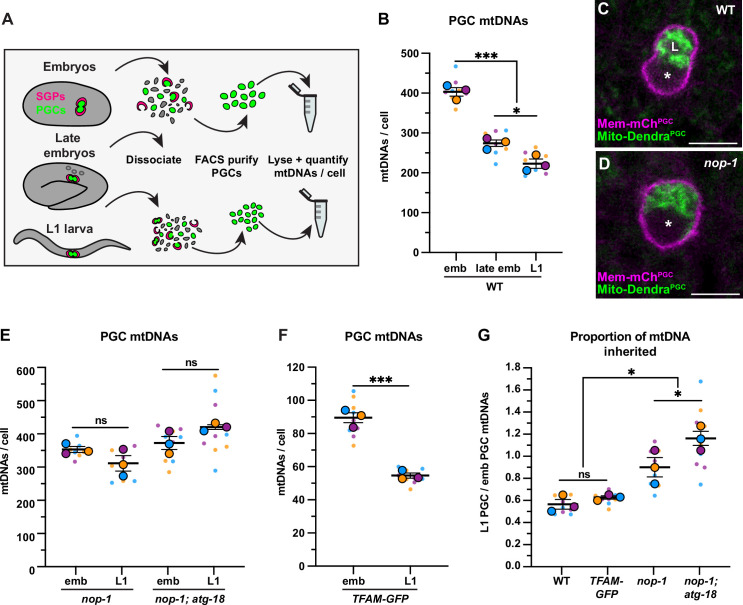


The originally published Figure 2 is shown for reference:

**Figure 6:**
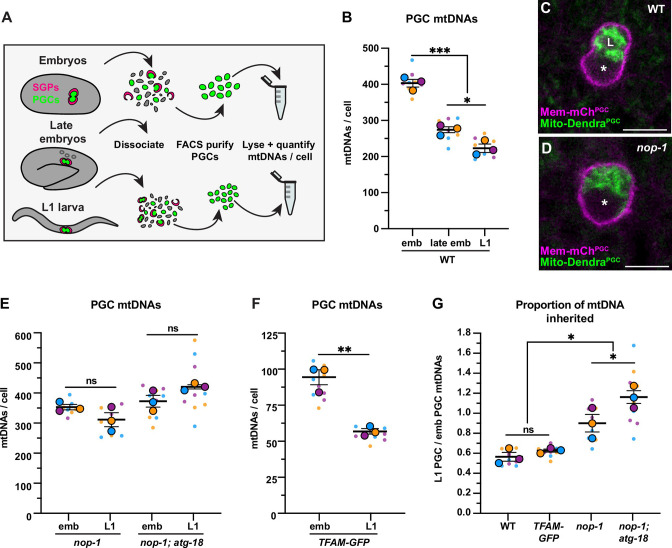


The article has been corrected accordingly.

