# Supplementary material for: Independent regulation of mitochondrial DNA quantity and quality in Caenorhabditis elegans primordial germ cells
Source: eLife. 2022 Oct 6;11:e80396. doi: 10.7554/eLife.80396 (PMC9536838; doi:10.7554/eLife.80396)
Supplement: Supplementary file 1. [file elife-80396-supp1.docx]

**Supplemental sequences**

***hmg-5(xn107[hmg-5-GFP]):***

Blue text = endogenous *hmg-5* locus (PAM edited),

UPPER CASE (exons), lower case (introns)

Gray text = Gly-Pro linker

Green text = GFP insertion sequence

UPPER CASE (exons), lower case (introns)

Red text = Stop, followed by 3' sequence

ATGTTGGGAACAATTTCAATGAGATTCTTCGCTACGAAAGTAGTTGCTCCACGTGCTTCTGTCGCAGCTTCAACTCCACAAGTCCCTCTTGGAATGAATATCAATCCATACGCAATGTTCATCAAAGAAAACTTCAAAGCTAACACTTCCGATATGAAGAGAACTGATTTGATGAAAGAGTTGTCTGGAAAGTGGAAGGCATTGAGTATCTCTGAAAAAGATgtaattataaatatagtttctaaaactaggataataaattatatttattgcagAAGTACACAGAACTCTCGAAAAATTACAATGCTCAAAAGCTGGATGACTTCATGAAACTATCTACTGAGGAACAGAAAAAATTGGTGGATTCTGCAAAAGAAAAGAAAGCGGAAAGAGCAAGTAGACGCCACGCAAAGGAACGCCGTGAAAAAAGGAAGCAATCTGGACGTCCAAGTGTTCCTCCAAGTGCTTATGCACTATTTATCAAAGAGAAGTTGTCTGGAGCTGGAATGGAATCCAAGGAGAAAATGAAAGAAGCTGTTGCTCAATGGAAGGCATTCACTGATTCCCAGAAAAAGgtaatatcagttttcgatttttcgaaaaaaaaaagctttaaaattaaaaaaatatattttgcttattgttgtattctgcacaaaaaaaaccaaaaccaaggctttattgaatagtagagagagtactcgataggacttaaggaataaacaaaaacataaagttcaattacgcatcgacttcaatatattttgcttattttcgtcttctcataaaaaattgtaggtgttattgtgttctttctcagcaactaaatttgataaaaattgagttttcgttataattaaaattttcaaacttattttttgattttccggagaatcaaaaaatcgaaattttttcgatgacacctttggtactcgataatatcaaatgattagacagtatttatctaggttaatgaattttagttatcaacaaaattcaacaataacgtttaaattaattttaataattttctttacagAAGTACACAGACGAAGCGAAGAAGCTGAAAGATGAATACCATGTCGTCCTCCAGAAATGGGAAGCAGAACAAAAAGAAAATGCAGATCAAGGGCCCATGAGTAAAGGAGAAGAACTTTTCACTGGAGTTGTCCCAATTCTTGTTGAATTAGATGGTGATGTTAATGGGCACAAATTTTCTGTCAGTGGAGAGGGTGAAGGTGATGCAACATACGGAAAACTTACCCTTAAATTTATTTGCACTACTGGAAAACTACCTGTTCCATGGgtaagtttaaacatatatatactaactaaccctgattatttaaattttcagCCAACACTTGTCACTACTTTCTgTTATGGTGTTCAATGCTTcTCgAGATACCCAGATCATATGAAACgGCATGACTTTTTCAAGAGTGCCATGCCCGAAGGTTATGTACAGGAAAGAACTATATTTTTCAAAGATGACGGGAACTACAAGACACgtaagtttaaacagttcggtactaactaaccatacatatttaaattttcagGTGCTGAAGTCAAGTTTGAAGGTGATACCCTTGTTAATAGAATCGAGTTAAAAGGTATTGATTTTAAAGAAGATGGAAACATTCTTGGACACAAATTGGAATACAACTATAACTCACACAATGTATACATCATGGCAGACAAACAAAAGAATGGAATCAAAGTTgtaagtttaaacatgattttactaactaactaatctgatttaaattttcagAACTTCAAAATTAGACACAACATTGAAGATGGAAGCGTTCAACTAGCAGACCATTATCAACAAAATACTCCAATTGGCGATGGCCCTGTCCTTTTACCAGACAACCATTACCTGTCCACACAATCTGCCCTTTCGAAAGATCCCAACGAAAAGAGAGACCACATGGTCCTTCTTGAGTTTGTAACAGCTGCTGGGATTACACATGGCATGGATGAACTATACAAATAGttgaattcgtcacaactttccattttgtaatcaaatatatcacttttcgattcatgacacttttctcaactacctcttctcaaatgtttcggttttatgtaaaatttatcgtatccc

***hmg-5(xn168[hmg-5-GFP_11_]):***

Blue text = endogenous *hmg-5* locus (PAM edited),

UPPER CASE (exons), lower case (introns)

Gray text = Gly-Gly-Ser-Gly-Gly linker

Green text = GFP_11_ insertion sequence

UPPER CASE (exons), lower case (introns)

Red text = Stop, followed by 3' sequence

ATGTTGGGAACAATTTCAATGAGATTCTTCGCTACGAAAGTAGTTGCTCCACGTGCTTCTGTCGCAGCTTCAACTCCACAAGTCCCTCTTGGAATGAATATCAATCCATACGCAATGTTCATCAAAGAAAACTTCAAAGCTAACACTTCCGATATGAAGAGAACTGATTTGATGAAAGAGTTGTCTGGAAAGTGGAAGGCATTGAGTATCTCTGAAAAAGATgtaattataaatatagtttctaaaactaggataataaattatatttattgcagAAGTACACAGAACTCTCGAAAAATTACAATGCTCAAAAGCTGGATGACTTCATGAAACTATCTACTGAGGAACAGAAAAAATTGGTGGATTCTGCAAAAGAAAAGAAAGCGGAAAGAGCAAGTAGACGCCACGCAAAGGAACGCCGTGAAAAAAGGAAGCAATCTGGACGTCCAAGTGTTCCTCCAAGTGCTTATGCACTATTTATCAAAGAGAAGTTGTCTGGAGCTGGAATGGAATCCAAGGAGAAAATGAAAGAAGCTGTTGCTCAATGGAAGGCATTCACTGATTCCCAGAAAAAGgtaatatcagttttcgatttttcgaaaaaaaaaagctttaaaattaaaaaaatatattttgcttattgttgtattctgcacaaaaaaaaccaaaaccaaggctttattgaatagtagagagagtactcgataggacttaaggaataaacaaaaacataaagttcaattacgcatcgacttcaatatattttgcttattttcgtcttctcataaaaaattgtaggtgttattgtgttctttctcagcaactaaatttgataaaaattgagttttcgttataattaaaattttcaaacttattttttgattttccggagaatcaaaaaatcgaaattttttcgatgacacctttggtactcgataatatcaaatgattagacagtatttatctaggttaatgaattttagttatcaacaaaattcaacaataacgtttaaattaattttaataattttctttacagAAGTACACAGACGAAGCGAAGAAGCTGAAAGATGAATACCATGTCGTCCTCCAGAAATGGGAAGCAGAACAAAAAGAAAATGCAGATCAAGGAGGTTCAGGAGGACGTGACCACATGGTCCTCCATGAGTATGTCAATGCCGCCGGAATCACCTAGttgaattcgtcacaactttccattttgtaatcaaa

***pink-1(xn199[pink-1(STOP-IN)])*:**

Blue text = endogenous *pink-1* locus (PAM edited),

UPPER CASE (exons), lower case (introns)

Red text = STOP-IN cassette

ATGTCTATGAAACGATTCGGAAAAGCAGCATATCGAATCGCAAATGAGgtatcttcaaaatcgttcttttttataatcgttaaattgaaattttttagTTAGTTGCAAAAGGTGGACGACTACCAATTTTCCAACGCTTCCTGCCGAGAATATTTCCCGCCACTTAGGAGCATCGGGAGCCTCAGGAGCATCGGGAGGTTCAGGAGGAGGGAAGTTTGTCCAGAGCAGAGGTGACTAAGTGATAAGCTAGCTAATTTAGGAGTTCATGTCGTACTCAAAAAGGCTCCATTTCCACGACAAAATGCTCTACGAATTgtaagtttatgaaaattcaacaaaaaaaaattaaaattgaattcctttcagGCTCGCCTTGTAACTCGCCACGGTCGAGTTTTCCGGCCATTTTCCTCAGTAATAATCGAAAGACATCGATTTCAAAATCAAAATGATTGGCGTCGAAAGTTTCAACCGATTCGTAAAGAATTGCCAAGAAATGTGGATTTAGTCGAACGAATCAGGCAGATATTTGGCAATTCTCTACGATACAATGAGGATTTGAAAAGCACTGAATGGCCGAATAGAATTGATTCTTATGAGTTTGgtatgcttttttcagtgatatttctccattgtttgagttttcagGGGAATTTCTCGGTCAAGGATGCAATGCAGCAGTTTACTCTGCGAGATTAGCCAATTCTGATGCAGAATCCTCAGGGAATACTCACTATGGTGCAGGGTTTAATGAAGTCACAAATATACTTGCAGAAATTCCGCCAGTTAGCAAAGTTGCACAAAAGgtagttgataatcttaattcgatgattaatattgaaaaatcattgcagAAATTCCCGTTGGCAATCAAATTAATGTTTAATTTTGAACATGATCGCGATGGAGATGCTCATCTCTTGAAATCAATGGGAAATGAATTGGCTCCATATCCGAATGCTGCAAAGTTGCTCAATGGACAAATGGGAACATTTAGACCTCTTCCAGCAAAACATCCAAATGTTGTTCGAATTCAGACAGCTTTTATTGATTCGTTAAAAGTTTTGCCAGATGCGATTGAACGgttagctttgaaatttattgataatgattgagaataagattttccagATATCCAGATGCCCTTCACACTGCACGTTGGTATGAGTCAATTGCCTCCGAACCGAAAACAATGTACGTAGTAATGAGACGATACCGACAAACACTTCATGAATATGTATGGACTCGTCATCGAAATTATTGGACAGGACGAGTGATAATTGCTCAACTATTAGAAGCATGTACATATCTTCATAAGCATAAAGTTGCTCAGCGAGACATGAAAAGTGATAATATTCTTCTGGAATATGATTTTGACGACGAGATTCCCCAATTAGTTGTCGCCGATTTTGGATGTGCACTTGCATGTGACAATTGGCAAGTAGACTATGAATCAGATGAAGTTAGTCTTGGAGGAAATGCCAAGACAAAAGCACCAGAAATTGCGACGGCTGTTCCTGGAAAGAATGTgtatgttttgaagtttagagtactgtatataatcaaagtttttacagAAAAGTAAACTTCGAAATGGCAGATACATGGGCAGCTGGAGGCCTTTCTTATGAAGTTCTAACACGATCAAATCCATTCTACAAACTTCTTGATACTGCAACATACCAGGAATCAGAACTACCAGCACTCCCATCTCGTGTCAATTTTGTGGCACGAGATGTCATTTTTGACCTACTCAAGCGAGATCCTAATGAAAGAGTCAAGCCGAATATTGCTGCAAATGCGTTGAATTTGTCATTGTTCAGAATGGGAGAAGATGTGAAGCAGATGATGGAAAAATGTGGAATATCTCAAATGACTACTCTATTGGCTGGAAGTTCTAAAGTTTTGAGTCAAAAAATCAATAGTCGTCTGGACAAAGTGATGAATCTGATTACTGCTGAAACTATCATGGCCAACCTAGCTCCACATTTGATTAGTCGAGCAGAACGACAACTTCGAGCAACATTTCTTTCAAGAATGAATCGAGAAGATATTTGGAGAAGTCTTCAATATTTCTTCCCAGCTGGTGTTCAACTTGACACACCTGCCACATCATCAGACTGTTTGGAGACTATTTCCAGTTTGATGTCGAGTTTTTCAAATGATTCAGAAAATTACGAGAAGCAACAGAAACCGGCTAAAAATGGATACAACAATGTTCCACTTCTTCTCAGAAATGTTATCCGTACAGATGCGGATGGAATCAATGGAATTGTACATAGAGTTCGATCTAAATAG

***dct-1(xn192[dct-1(STOP-IN)])*:**

Blue text = endogenous *dct-1* locus (PAM edited),

UPPER CASE (exons), lower case (introns)

Red text = 3xHA-STOP-IN cassette

ATGTCCTCATTTCTTGAGTTTGCAAAACCCAAAATGCTCGATATCAAAAGAAAGATCAACTTTGCGTCCGGCGAGAAGACGGATGAGTCTGTGCAACCGCAACAGCAGACGGAGCAGTCATCGGCTCAACAGACAACACCATCCGCAAAAGCCGTCTACCCATACGACGTGCCAGACTATGCATATCCATACGACGTTCCAGACTACGCTTATCCATATGATGTTCCAGATTATGCTTGACTAAGTGATAAGCTAGCTTGACCGAGAGTACACCTGgtttgttttacttttttttcattacacttttgccctaggcaaatcgttagatgaattctcatgtgaccgattgggcttttgattttttgaaagtacacacatctttgcaagtttacaaatttaacacctattcacttttcactaactccaacttaaatttcccatgttcttgaacgcttcagGTATGTCAGAATCGTGGGTGGAACTGGCACCAAGCCGTACCAGTTTGTGCAGTAGCGTCGATATTAATATGGTGATCATTGATGAAAAAGATAAGGATTCGAGGCTGAGCCCAGTCTCAATCGCACAATCTCCTCACGTGGAATTCGAGAGCTTAGAACAAGTAAAGTACAAGTTGGTTAGAGAAATGCTTCCTCCCGGAAAGAATACTGATTGGATTTGGGATTGGAGCAGTCGGCCAGAAAATACACCTCCAAAgtaagttttatagctactaatgggttgaaagaagcttagttatttagacttcaaaattaatttagaaccagggatgcgctgcatttgctaaccagcagttctacaaaataagccagcatggcatgttctcaaaataattctaaaaaacgcagatacattgttgaaagattaaaactgctagagaaaagttacatagtttgtcggactattttcagtaattttcaactttcaatctaaactaaaatatgtttgctaattgaaaattttctaaaagagattttttgaaaatacttttatattccatacctaaatgttactgaaaatattggaagaacatgttgcaaggttcaatttgtagaattgctagtcgacacccctgctcaaaccataccgattttattctgtgttgaaaaaaaaaacaaaatttgcagGACTGTCCGTATGGTTCAATATGGCTCCAACCTTACCACTCCGCCAAACTCTCCCGAGCCAGAGCTCTACCAATACTTGCCATGCGAATCTGATTCGCTTTTCAACGTTCGCGTAGTCTTTGGTTTCTTAGTCACCAACATCTTCAGCTTTGTCGTTGGAGCAGCAGTAGGgtaagtatttttttcaggaaatttcttccagaaaaactgattgtcttcagATTTGCTGTTTGCCGGAAACTAATCAAACATCATCGTCAATAA

***GFP_1-10_:***

Green text = GFP_1-10_ sequence used

UPPER CASE (exons), lower case (introns)

Red text = Stop

ATGTCTAAGGGAGAAGAATTATTTACTGGAGTTGTTCCTATCCTCGTCGAGCTCGACGGAGACGTCAACGGACACAAGTTCTCCGTCCGTGGAGAGGGAGAGGGAGACGCCACCATTGGAAAGCTCACCCTCAAGTTCATCTGCACCACCGGAAAGCTCCCAGTCCCATGGCCAACCCTCGTCACCACCCTCACCTACGGAGTCCAATGCTTCTCCCGTTACCCAGACCACATGAAGAGACACGACTTCTTCAAGTCCGCCATGCCAGAGGGATACGTCCAAGAGCGTACCATCTCCTTCAAGgtaagtttaaacatatatatactaactaaccctgattatttaaattttcagGACGACGGAAAATACAAGACCCGTGCCGTTGTCAAGTTCGAGGGAGACACCCTCGTCAACCGTATCGAGCTCAAGgtaagtttaaacagttcggtactaactaaccatacatatttaaattttcagGGAACAGACTTCAAGGAGGACGGAAACATCCTCGGACACAAGCTCGAGTACAACTTCAACTCCCACAACGTCTACATCACCGCCGACAAGCAAAAGAACGGAATCAAGGCCAACTTCACAgtaagtttaaacatgattttactaactaactaatctgataataattttcagGTTCGTCACAACGTCGAGGACGGATCCGTCCAACTCGCCGACCACTACCAACAAAACACCCCAATCGGAGACGGACCAGTCCTCCTCCCAGACAACCACTACCTCTCCACCCAAACAGTTCTCTCCAAGGACCCAAACGAGAAGTAA

***mito(matrix)GFP_1-10_:***

Yellow text = mitochondrial matrix localization sequence

Green text = GFP_1-10_ sequence used, PAM edited

UPPER CASE (exons), lower case (introns)

Red text = Stop

ATGGCACTCCTGCAATCACGTCTCCTCCTGTCCGCCCCACGTCGTGCCGCCGCCACCGCCCGTGCCGGAGCTGGTGCAGGCGCTGGAGCCGGAGCCATGTCTAAGGGAGAAGAACTCTTCACTGGAGTTGTTCCTATCCTCGTCGAGCTCGACGGAGACGTCAACGGACACAAGTTCTCCGTCCGTGGAGAGGGAGAGGGAGACGCCACCATTGGAAAGCTCACCCTCAAGTTCATCTGCACCACCGGAAAGCTCCCAGTCCCATGGCCAACCCTCGTCACCACCCTCACCTACGGAGTCCAATGCTTCTCCCGTTACCCAGACCACATGAAGAGACACGACTTCTTCAAGTCCGCCATGCCAGAGGGATACGTCCAAGAGCGTACCATCTCCTTCAAGgtaagtttaaacatatatatactaactaaccctgattatttaaattttcagGACGACGGAAAATACAAGACCCGTGCCGTTGTCAAGTTCGAGGGAGACACCCTCGTCAACCGTATCGAGCTCAAGgtaagtttaaacagttcggtactaactaaccatacatatttaaattttcagGGAACAGACTTCAAGGAGGACGGAAACATCCTCGGACACAAGCTCGAGTACAACTTCAACTCCCACAACGTCTACATCACCGCCGACAAGCAAAAGAACGGAATCAAGGCCAACTTCACAgtaagtttaaacatgattttactaactaactaatctgataataattttcagGTTCGTCACAACGTCGAGGACGGATCCGTCCAACTCGCCGACCACTACCAACAAAACACCCCAATCGGAGACGGACCAGTCCTCCTCCCAGACAACCACTACCTCTCCACCCAAACAGTTCTCTCCAAGGACCCAAACGAGAAGTAA

***mito-tomm-20^1-54^-Dendra2:***

Yellow text = *tomm-20* mitochondrial outer membrane localization sequence

Gray text = NPAFLYGS linker

Green text = Dendra2 sequence used

UPPER CASE (exons), lower case (introns)

Red text = Stop

ATGTCGGACACAATTCTTGGTTTCAACAAATCAAACGTCGTTTTGGCTGCTGGAATTGCTGGAGCCGCTTTCCTCGGCTACTGCATTTACTTCGATCATAAGAGAATCAACGCTCCAGACTACAAGGACAAGATTAGGCAAAAGAGACGTGCCCAGGCTGGAAACCCAGCTTTCTTGTACGGATCCATGAACCTTATTAAGGAAGATATGAGAGTCAAAGTTCATATGGAAGGAAACGTCAACGGTCATGCATTTGTTATTGAAGGAGAAGGAAAAGGAAAGCCATACGAAGGAACTCAAACTGCAAACTTGACTGTCAAAGAAGGAGCACCACTACCATTTAGTTACgtaagtttaaacatatatatactaactaaccctgattatttaaattttcagGATATTCTAACTACTGCCGTCCATTACGGAAACAGAGTTTTTACTAAATACCCAGAAGATATTCCTGATTACTTCAAGCAATCGTTTCCAGAAGGATACTCGTGGGAAAGAACTATGACTTTCGAAGATAAAGGTATTTGCACTATTgtaagtttaaacagttcggtactaactaaccatacatatttaaattttcagAGAAGTGATATTAGTCTAGAAGGTGATTGCTTCTTCCAAAATGTCAGATTTAAAGGAACTAACTTTCCTCCTAACGGACCAGTTATGCAAAAGAAGACTCTTAAGTGGGAACCATCGACTGAAAAACTACATGTTAGAGATGGACTACTTGTTGGAgtaagtttaaacttggacttactaactaacggattatatttaaattttcagAACATTAACATGGCACTACTACTAGAAGGTGGAGGTCACTACCTTTGCGATTTTAAAACTACTTACAAAGCAAAGAAGGTCGTCCAACTTCCAGATGCACACTTTGTTGATCACAGAATTGAAATACTAGGAAACGATTCGGATTACAACAAAGTTAAGCTATACGAACACGCAGTTGCAAGATACAGTCCTCTACCAAGTCAAGCATGGTAA
